# Supplementary material for: A versatilely high fidelity electric machines emulator for rapid testing of motor controller
Source: PLoS One. 2024 May 2;19(5):e0299371. doi: 10.1371/journal.pone.0299371 (PMC11065206; doi:10.1371/journal.pone.0299371)
Supplement: Attachment — Submitted filename: Response to Reviewers.docx [file pone.0299371.s009.docx]

**Manuscript Number: PONE-D-23-24308**

**A Versatilely High Fidelity Electric Machines Emulator for Rapid Testing of Motor Controller**

Yifeng Guo^1^, Limin Huang^1^*, Min Zhang^2^, Min He^2^, Bin Zhong^1^, Dakun Fan^1^

**Dear Reviewer and Editor:**

Thank you for your helpful comments and suggestions on our manuscript. We have modified the manuscript accordingly. The main changes to the original submission, made in response to these comments and suggestions, are shown in **blue** color in the revised version. We present here the detailed response to the reviewer’s comments on our work point by point as below:

**Response to Reviewer #1:**

**Comment 1 and Comment 2:**

1. It is hard to recognize any novelty in presented work. This is because; operation of an electrical machine with their non-linear characteristics has been already reported in many available literatures.

2. Furthermore, Real-time simulation using hardware emulator is also reported [1, 8, 11, 17, 18, 20].

**Response 1 and 2:**

The objective of this study is to develop a simulator that can simulate the behavior of a motor in real time by combining the proposed motor model with typhoon. The simulator serves as a control object for the motor controller and helps the developer to develop the related control program in advance. Although many methods for describing motor nonlinearities have been reported in existing studies, the magnetic chain-based motor modeling method proposed in this paper characterizes the real operating characteristics of IPM motors, improves the simulation accuracy and efficiency of the model motor model, and the method has low computational complexity and high real-time performance. The magnetic chain-based motor model can be deployed on in the semi-physical simulation equipment with the advantages of stability and high reliability.

**Comment 3:**

3. HIL results in the manuscripts seem to be obtained from PC simulation using Typhoon software. It should be captured using suitable indicating instrument/DSO.

**Response 3:**

The result graph is drawn from the data exported by the oscilloscope through matlab, and no screenshots of the oscilloscope are retained during the experimental phase. The resulting graph proves that the simulator accurately represents the real behavior of the motor, and we will incorporate your suggestions in subsequent studies.

**Comment 4:**

Authors are required to mention the suitable reason for the consideration of Interior Permanent Magnet (IPM) machine in their work.、

**Response 4:**

The detailed parameters of the IPM motor in this study come from Toyota's open source motor data, and IPM is widely used in electric vehicles, so this is the motivation for choosing the IPM motor in this article. We will redescribe this part in Chapter 3 of the paper.

**Comment 5:**

Authors are also required to add more recent and relevant literature in reference section.

**Response 5:**

We have added some recent references to the paper.

**Response to Reviewer #2:**

**Comment 1:**

1. In the Steady State Simulation Analysis section I suggest summarizing the results in a table.

**Response 1**

We summarize the results of the steady-state simulation in Table 2.

**Comment 2:**

2. In the Electric Machines Emulator Transient Simulation Analysis section I suggest summarizing the results in a table.

**Response 2**

We summarize the results of the transient -state simulation in Table 3.

**Comment 3:**

3. Improving the quality of the figures in the article.

**Response 3**

We have redrawn the experimental results to improve picture clarity.
